# Long-term outcomes after coronary artery bypass surgery in patients with rheumatoid arthritis

**DOI:** 10.1080/07853890.2021.1969591

**Published:** 2021-08-31

**Authors:** Markus Malmberg, Antti Palomäki, Jussi O. T. Sipilä, Päivi Rautava, Jarmo Gunn, Ville Kytö

**Affiliations:** a Heart Center, Turku University Hospital and University of Turku, Turku, Finland; b Centre of Rheumatology and Clinical Immunology, Division of Medicine, Turku University Hospital, Turku, Finland; c Department of Medicine, University of Turku, Turku, Finland; d Institute for Molecular Medicine Finland, FIMM, HiLIFE, University of Helsinki, Helsinki, Finland; e Department of Neurology, North Karelia Central Hospital, Siun Sote, Joensuu, Finland; f Clinical Neurosciences, University of Turku, Turku, Finland; g Department of Public Health, University of Turku, Turku, Finland; h Turku Clinical Research Centre, Turku University Hospital, Turku, Finland; i Research Center of Applied and Preventive Cardiovascular Medicine, University of Turku, Turku, Finland; j Center for Population Health Research, Turku University Hospital and University of Turku, Turku, Finland; k Administrative Center, Hospital District of Southwest Finland, Turku, Finland

**Keywords:** Case-control study, coronary artery disease, coronary artery bypass grafting surgery, outcomes, rheumatoid arthritis

## Abstract

**Objective:**

To investigate the long-term outcomes of coronary artery bypass grafting surgery (CABG) in patients with rheumatoid arthritis (RA).

**Methods:**

Patients with RA (*n* = 378) were retrospectively compared to patients without RA (*n* = 7560), all treated with CABG in a multicentre, population-based cohort register study in Finland. The outcomes were studied with propensity score-matching adjustment for baseline features. The median follow-up was 9.7 years.

**Results:**

Diagnosis of RA was associated with an increased risk of mortality after CABG compared to patients without RA (HR 1.50; CI 1.28–1.77; *p* < .0001). In addition, patients with RA were in higher risk of myocardial infarction during the follow-up period (HR 1.61; CI 1.28–2.04; *p* < .0001). Cumulative rate of repeated revascularization after CABG was 14.4% in RA patients and 12.0% in control patients (*p* = .060). Duration of RA before CABG (*p* = .011) and preoperative corticosteroid usage in RA (*p* = .041) were independently associated with higher mortality after CABG. There were no differences between the study groups in 30-d mortality or in the post-operative usage of cardiovascular medications.

**Conclusions:**

RA is independently associated with worse prognosis in coronary artery disease treated with CABG. Preoperative corticosteroid use and longer RA disease duration are additional risk factors for mortality.

**Key messages**
- Patients with rheumatoid arthritis (RA) have impaired long-term outcomes after coronary artery bypass surgery (CABG).
- Glucocorticoid use before CABG and duration of RA are associated with higher mortality.
- Special attention should be paid in secondary prevention of cardiovascular disease in RA patients after CABG.

## Introduction

Patients with rheumatoid arthritis (RA) have a significantly increased risk for coronary artery disease compared with the general population [1,2] and coronary artery disease is a major cause for increased mortality in these patients [3,4]. Coronary revascularization is often needed in the treatment of coronary artery disease also in RA patients [5]. Previous studies have shown that RA is associated with increased mortality and morbidity after percutaneous coronary intervention (PCI) [6]. The outcomes after coronary artery bypass crafting surgery (CABG) in RA patients are not sufficiently studied. In previous reports, the number of patients with RA has been relatively small [7,8] or the studies have been focussed mainly on in-hospital outcomes [9,10]. In this study, we investigated the long-term outcomes of RA patients treated with CABG using in a large nationwide registry.

## Patients and methods

### Study design

We studied the outcome of RA patients after CABG for coronary artery disease. Propensity score-matching was used to balance baseline differences between study groups. The primary outcome of interest was mortality. Secondary outcomes were myocardial infarction, revascularization and post-operative usage of cardiovascular medication.

### Study population

**Figure 1.**
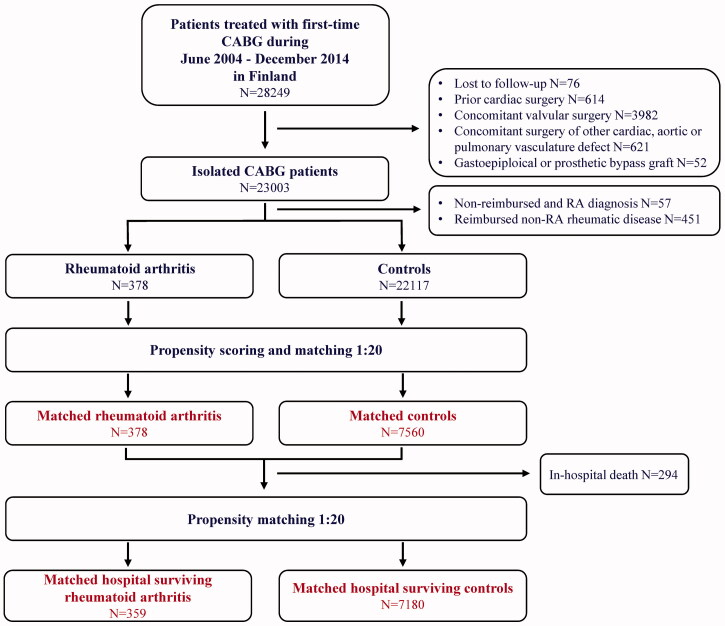
Study flow-chart. CABG: coronary artery bypass grafting surgery; RA: rheumatoid arthritis.

Patients treated with first-time CABG between 1 June 2004 and 31 December 2014 (*n* = 28,249) were retrospectively recognized from The Care Register for Healthcare in Finland. This mandated by law, nationwide registry includes data on all hospital admissions and major surgical procedures in Finland [11]. CABG was performed in eight hospitals (six public and two private) during the study period. Patients with prior cardiac surgery, concomitant surgery of heart valves, aorta, ventricles, atria or other cardiac or pulmonary vasculature defects, as well as bypass surgery using gastroepiploic arterial or prosthetic grafts, or unavailable follow-up of surviving patients (*n* = 76, mostly foreign nationals) were excluded from this study. Patients with RA were recognized from the database using ICD-10 codes M05 and M06 for seropositive and seronegative RA, respectively. In Finland, all patients with appropriately diagnosed RA are entitled to special reimbursement for medication. To gain this reimbursement, a rheumatologist or a doctor working at a rheumatology clinic writes a medical certificate describing the rationale for the diagnosis, and the certificate is then delivered to the Social Insurance Institute of Finland. To ensure that the diagnoses of RA were correct, only patients with the special reimbursement for antirheumatic medications were included in this study, resulting altogether 378 patients with and 22117 without RA (Figure 1).

### Definitions

Co-morbidities were recognized from the Care Register for Healthcare, the Finnish Cancer Registry, and the Nationwide database of permissions for drug reimbursements in Finland using previously described ICD coding [12] and applicable drug purchase reimbursement codes (https://www.kela.fi/web/en/medicine-expenses). Myocardial infarctions (ICD-10 code I21* or I22*) were detected from admission records and death certificates. Drug purchases were recognized using applicable ATC-coding (Supplement Tables 1 and 2). Pre-operative usage of per oral corticosteroids was defined as purchase within 6 months prior to CABG. Post-operative cardiovascular drug usage was defined as new drug purchase within 90 d after initial discharge from CABG. Duration of RA was defined as time from reimbursement permission to CABG. Drug purchase data (including ATC-codes and purchase dates) and drug purchase reimbursement permission data were obtained from nationwide registry held by the Social Insurance Institution of Finland. Mortality data were obtained from nationwide cause of death registry held by Statistics Finland. These registries are mandated-by-law and have a full coverage of drug purchases, reimbursement permissions, and deaths in Finnish population. Follow-up ended on 31 December 2018. The study was approved by the National Institute for Health and Welfare of Finland (permission no: THL/2245/5.05.00/2019), Statistics Finland (TK-53-484-20), and the Social Insurance Institution of Finland (91/522/2015). This was a retrospective register study, and thus informed consent was waived, and the participants were not contacted.

**Table 1.** Baseline features of patients treated with isolated coronary artery bypass surgery.

	Original cohort				
	Rheumatoid arthritis	Control			Matched cohort
Variable	*N* = 378	*N* = 22,117	*p* Value	|SMD|	|SMD|
Age, years (SD)	67.9 (8.6)	66.7 (9.2)	.001	0.14	0.03
Female sex	133 (35.2%)	4746 (21.5%)	<.0001	0.31	0.004
Co-morbidities					
Atrial fibrillation	52 (13.8%)	2278 (10.3%)	.029	0.11	0.001
Cerebrovascular disease	31 (8.2%)	1720 (7.8%)	.760	0.02	0.01
Chronic pulmonary disease	65 (17.2%)	2289 (10.4%)	<.0001	0.20	0.01
Diabetes	95 (25.1%)	5871 (26.6%)	.537	0.03	0.02
Heart failure	79 (20.9%)	3288 (14.9%)	.001	0.16	0.02
Hypertension	220 (58.2%)	12066 (54.6%)	.158	0.07	0.004
Malignancy	26 (6.9%)	1641 (7.4%)	.690	0.02	0.003
Peripheral vascular disease	43 (11.4%)	1603 (7.2%)	.002	0.14	0.01
Psychotic disorder	5 (1.3%)	427 (1.9%)	.393	0.05	0.02
Prior myocardial infarction	97 (25.7%)	4715 (21.3%)	.041	0.10	0.004
Renal disease	22 (5.8%)	420 (1.9%)	<.0001	0.20	0.05
Myocardial infarction^a^	31 (8.2%)	1718 (7.8%)	.755	0.02	0.01
Type of bypass graft			.191	0.13	0.001
Only arterial	62 (16.4%)	3303 (14.9%)			
Only venous	28 (7.4%)	1230 (5.6%)			
Arterial and venous	288 (76.2%)	17548 (79.5%)			
Number of grafted anastomoses			.690	0.05	0.003
1	44 (11.6%)	2111 (9.5%)			
2	49 (13.0%)	2968 (13.4%)			
3	120 (31.8%)	6875 (31.1%)			
4	102 (27.0%)	6312 (28.5%)			
≥5	63 (16.7%)	3815 (17.4%)			
Surgical centre (*n* = 8)			.013	0.27	0.05
Year of surgery			.429	0.14	0.04

Differences between groups for all and for propensity score-matched patients.

^a^As indication for surgery. Matched (1:20) cohort includes all 378 patients with rheumatoid arthritis and 7560 controls.

SMD: Standardized mean difference.

**Table 2.** Association of seropositivity, corticosteroid usage and disease duration with long-term mortality after coronary artery bypass in rheumatoid arthritis.

	Univariable		Model 1		Model 2	
Variable	HR (95% CI)	*p* Value	HR (95% CI)	*p* Value	HR (95% CI)	*p* Value
Seropositivity	1.16 (0.79–1.70)	.448	1.03 (0.69–1.57)	.874	0.87 (0.57–1.33)	.415
Per oral corticosteroid usage	1.48 (1.08–2.02)	.014	1.44 (1.03–2.01)	.032	1.42 (1.01–1.99)	.041
Duration of RA (per 5-year increment)	1.02 (1.01–1.03)	.003	1.09 (1.02–1.17)	.009	1.10 (1.02–1.17)	.011

Model 1 was adjusted for age, sex, atrial fibrillation, cerebrovascular disease, chronic pulmonary disease, diabetes, heart failure, hypertension, malignancy, peripheral vascular disease, psychotic disorder, myocardial infarction (prior and acute), renal disease, type of bypass graft, number of grafted anastomoses and surgical centre.

Model 2 = Model 1 + seropositivity, corticosteroid usage and duration of RA.

HR: hazard ratio; RA: rheumatoid arthritis.

Results of all in included variables are presented in Supplement Table 3.

### Matching

Propensity score-based matching on baseline characteristics (Table 1) was created using logistic regression. Patients with non-overlapping propensity scores were excluded (*n* = 350 patients without RA). We performed a 1:20 optimal propensity score matching without replacement, with a calliper set at 0.1 times the standard deviation of the estimated propensity score [13]. Hospital survivors of initial matching were re-matched (1:20). The extent of unmeasured confounding was estimated by the E-value [14].

### Statistical analysis

Differences between groups were studied with t-test and chi-square test (non-matched groups) or with paired t-test and McNemar’s test (matched groups). Effect sizes in baseline characteristics between study groups were evaluated by standardized mean difference (SMD). Usage of cardiovascular medication after CABG was studied using logistic regression. Mortality, MI, and repeated revascularization were studied with Kaplan–Meier method and Cox regression. Regression models stratified for matching were used in analysis of propensity-matched groups. Association of seropositivity, pre-operative corticosteroid usage and duration of RA with long-term mortality were studied using Cox regression models (Table 2, Supplement Table 3). Visual examination of Schoenfeld residuals was used for confirmation of proportional hazard assumptions. Cause-specific hazard models were used. Median follow-up time of 9.7 years (min 4.0 and max 14.3) was calculated for survivors. Results are given as the mean, median, percentage, hazard ratio (HR) or odds ratio (OR) with 95% confidence intervals (CIs) or ± SD. *p* Value < .05 was considered statistically significant. Analyses were performed with SAS version 9.4 (SAS Institute Inc., Cary, NC).

**Table 3.** Post-discharge prescription medication in rheumatoid arthritis patients and matched controls treated with coronary artery bypass grafting.

	Rheumatoid arthritis	Matched controls		
	*N* = 359 (%)	*N* = 7180 (%)	OR (95% CI)	*p* Value
ADP-inhibitor	37 (10.3)	785 (10.9)	0.94 (0.66–1.33)	.709
Anticoagulant	70 (19.5)	1655 (23.1)	0.81 (0.62–1.06)	.117
Antidiabetic	78 (21.7)	1582 (22.0)	0.98 (0.76–1.27)	.891
Insulin	40 (11.1)	667 (9.3)	1.23 (0.87–1.73)	.238
Non-insulin	55 (15.3)	1182 (16.5)	0.92 (0.68–1.23)	.566
ACEi or ARB	188 (53.4)	4048 (56.4)	0.85 (0.69–1.05)	.136
Antiarrhythmic	12 (3.3)	276 (3.8)	0.87 (0.48–1.56)	.630
Beta-blocker	321 (89.4)	6401 (89.2)	1.03 (0.73–1.45)	.875
Ca-blocker	61 (17.0)	1203 (16.8)	1.02 (0.77–1.35)	.906
Digitalis	20 (5.6)	357 (5.0)	1.13 (0.71–1.79)	.611
Diuretic	181 (50.4)	3325 (46.3)	1.18 (0.96–1.47)	.124
Nitrate	86 (24.0)	1596 (22.2)	1.10 (0.86–1.41)	.443
Statin	306 (85.2)	6154 (85.7)	0.96 (0.71–1.30)	.802
Intensity of statin therapy				.847
Low	19 (6.2)	348 (5.7)	–	–
Moderate	244 (79.7)	4884 (79.4)	–	–
High	43 (14.1)	922 (15.0)	–	–

ADP: adenosine diphosphate; ACEi: angiotensin-converting-enzyme inhibitor; ARB: angiotensin receptor blocker; OR: odds ratio

## Results

Of all included CABG patients (median age 67 years, 21.7% women) 1.6% had RA with reimbursement permission assuring definite diagnosis. RA patients were older and had more co-morbidities than control CABG patients (Table 1). Atrial fibrillation, chronic pulmonary disease, heart failure, peripheral vascular disease, prior myocardial infarction and renal disease were more common in RA group. Type of bypass graft or number of coronary anastomoses did not differ between study groups (Table 1). Baseline differences were balanced by propensity matching resulting to 378 patients with RA and 7560 patients without RA (Table 1). Re-matching of hospital survivors resulted in 359 RA and 7180 control patients without baseline differences (SMD ≤ 0.06).

Majority of RA patients (79.1%) were seropositive. The median duration of RA before CABG was 12.0 years (IQR 5.4–22.5 years). An oral corticosteroid was used by 38.4% of RA patients before surgery.

### Mortality

**Figure 2.**
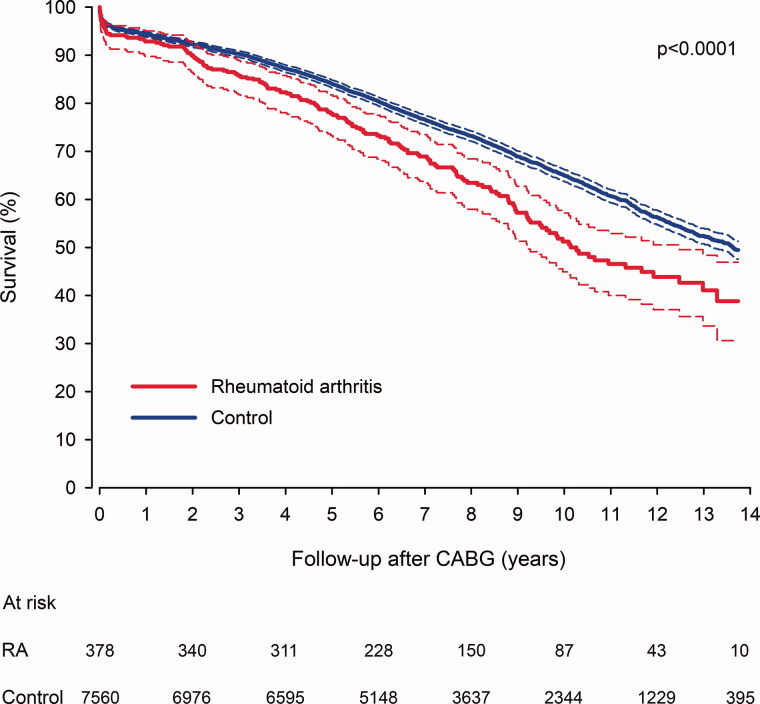
Survival in rheumatoid arthritis and matched control patients after coronary artery bypass surgery. CABG: coronary artery bypass grafting surgery; RA: rheumatoid arthritis.

During the follow-up there were a total of 2750 deaths (163 in RA group and 2587 in matched controls). Thirty-day mortality of operated patients was 3.7% in RA group *vs.* 2.9% in controls (*p* = .411). RA patients had higher mortality in long-term follow-up with the survival difference beginning to increase from 2-year follow-up onwards (Figure 2). The cumulative mortality rate at the end 14.3-year follow-up after CABG was 65.5% in RA patients and 54.4% in controls (HR 1.50; CI 1.28–1.77; *p* < .0001). The E-value was 2.37 (CI 1.88–2.93). Proportion of cardiovascular deaths was higher in RA (54.4%) compared to control (53.0%) patients (*p* < .0001). Duration of RA and preoperative corticosteroid usage, but not seropositivity was independently associated with higher long-term mortality in RA patients (Table 2).

### Myocardial infarction

**Figure 3.**
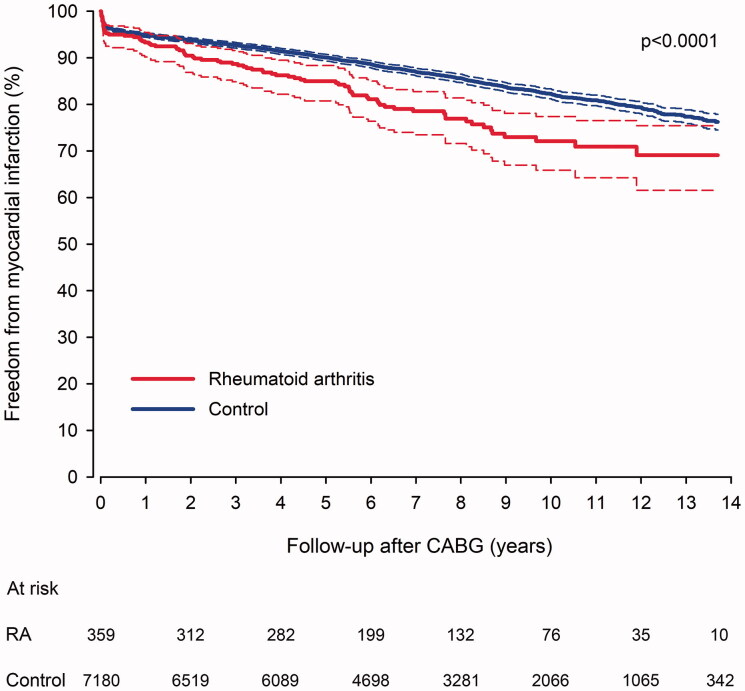
Freedom from myocardial infarction in rheumatoid arthritis and matched control patients after coronary artery bypass grafting. CABG: coronary artery bypass grafting surgery; RA: Rheumatoid arthritis.

Myocardial infarction occurred in 1190 patients (81 in the RA group) during the follow-up of hospital survivors. In RA patients, the cumulative rate of all myocardial infarcts was 7.1% at 1-year, 15.4% at 5-year, 28.2% at 10-year and 39.6% at 14.3-year follow-up (Figure 3). Corresponding occurrence of myocardial infarction in matched controls was 4.7% at 1-year, 8.8% at 5-year, 16.1% at 10-year and 25.2% at 14.3-year follow-up. Patients with RA had a higher hazard of myocardial infarction during the follow-up (HR 1.61; 1.28–2.04; *p* < .0001). Cumulative rate of myocardial infarction admission during follow-up was 37.2% in RA and 22.6% in control groups (HR 1.55; CI 1.21–1.99; *p* = .0005). Occurrence of fatal myocardial infarction was 15.0% in RA group and 7.2% in control group (HR 2.30; CI 1.48–3.57; *p* = .0002). First myocardial infarction after CABG was fatal more frequently in RA patients (19.2 *vs.* 18.0%, *p* < .0001) than in the control group.

### Repeated revascularization

**Figure 4.**
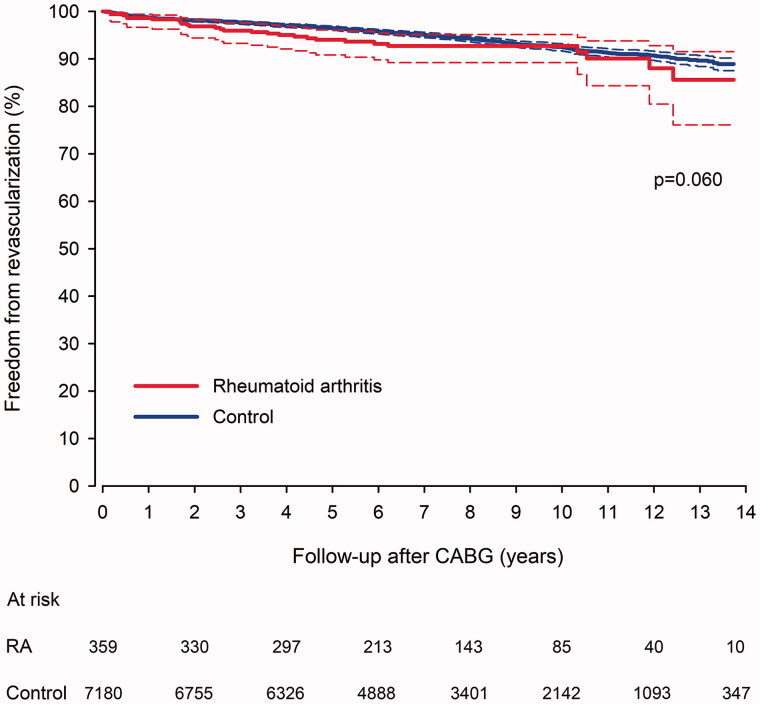
Freedom from repeated revascularization after coronary artery bypass surgery in rheumatoid arthritis and matched control patients. CABG: coronary artery bypass grafting surgery; RA: rheumatoid arthritis.

Of patients treated CABG, 475 (27 in RA group) were repeatedly revascularized during the follow-up. Cumulative rate of repeated revascularization after CABG was 14.4% in RA patients and 12.0% in controls (*p* = .060, Figure 4). First-line revascularization after CABG was PCI in 99.3% of revascularized patients. Redo CABG was rare during follow-up (0.0% in RA *vs.* 0.3% in control group, *p* = .995).

### Usage of cardiovascular medication

Post-discharge prescription medications for cardiovascular diseases after primary hospitalization for CABG are presented in Table 3. There were no significant differences between RA patients and matched control patients in drug usage or intensity of statin therapy.

## Discussion

Our multicentre nationwide retrospective cohort study shows that during a median follow-up 9.7 years, patients with RA had increased mortality and increased risk of myocardial infarction after CABG compared to matched controls. Risk of death was associated with longer duration of RA and preoperative use of oral corticosteroids.

Patients with RA are a unique high-risk group among coronary artery disease patients [1,2,15]. In a single-centre study by Spartera et al., the investigators studied the long-term outcomes of coronary artery disease in a cohort of RA patients, reporting that patients with RA had a higher rate of major adverse cardiovascular and cerebrovascular events after coronary revascularizations [5]. During a median follow-up of over 9 years, 47% of patients treated with a PCI had a repeat coronary revascularization and 30% of CABG patients had suffered a stroke. Similarly, Lai et al. reported that RA was associated with significantly increased risk of mortality and ischaemic complications in PCI patients [6]. However, in another study by the same group, they reported that RA was not associated with adverse outcomes after CABG compared to control group [8]. This finding contrasts our results and the difference might be, at least in part, explained by the differing sample size, since in our study there are over 3 times of RA patients than they reported. To our knowledge, our report is the first study to show, that patients with RA have impaired long-term outcome after CABG. Our results, therefore, bring important perspective to this matter.

Patients treated with CABG provide a cohort of widespread and severe coronary artery disease, providing insight into influence of RA to already advanced coronary artery disease. A recent study by Løgstrup et al. showed that patients with coronary angiography verified coronary artery disease, comorbid RA was associated with significantly higher risk of cardiovascular events and mortality [16]. Remarkably, there is some evidence indicating, that patients with RA might have better hospital survival when revascularized with PCI or CABG, even after suffering from myocardial infarction [9,10,17]. In our study, there were no differences between the study groups in 30-d mortality postoperatively. In previous studies, the excess mortality of patients with RA compared to general population has been shown to emerge approximately 10 years from diagnosis of RA, although the risk is highly dependent on disease activity [18]. It has been suggested, that disease activity over time might be the key driver of excess mortality compared to disease duration itself [19]. Our results indicate, that longer duration of RA was associated with higher mortality after CABG even in the multivariable analysis adjusted for age, sex and numerous competing risk factors for mortality, and the difference in mortality began to increase two years after CABG.

This study shows that preoperative glucocorticoid usage was a strong predictor of mortality during follow-up. Also previous studies have indicated, that both cumulative and average daily dose of glucocorticoids associate with increased risk of adverse cardiovascular events [20,21], and high doses are associated with increased mortality [22]. George et al. showed in their report that glucocorticoids were associated with an increased risk in 90-d mortality after cardiac surgery in dose-dependent manner [23]. Besides known detrimental effects of the medication itself, higher dose of glucocorticoids can also be a sign of more aggressive RA with high disease activity, potentially explaining part of the increased risk for cardiovascular events [24]. Evolvement of RA treatments, especially biological drugs, may possibly influence the outcomes and require further study.

In our study, only 85% of patients were on statin therapy after surgery and 14% received high-intensity statin therapy, recommended by the current the guidelines for high-risk patients with established coronary artery disease [25,26]. This finding could also affect long-term survival and cardiovascular outcomes investigated here.

This study has limitations. First, the retrospective design limits the possibility to interpret the results. We used combination of previously validated nationwide registries, which are mandated-by-law in Finland [27]. Although the data collected from the registries are reliable, it is, however, possible, that sources of bias are present. Diagnoses were determined by clinicians and coding errors are therefore possible. The incidence of RA patients in our cohort was 1.6% with definite diagnosis, which is comparable with previously reported (0.25–1.2%) [7–9,17]. We believe that the design and methodology in this study helped us to accurately identify all the patients with RA diagnosis treated with CABG. Second, the use of retrospective registries does not allow access to more detailed information regarding operative or in-hospital data, or detailed information of the RA disease activity. However, it is not expected that it could interfere with the essential findings of this study. Third, propensity score was used to balance differences in major risk factors between the study groups. It is possible that residual co-founders that where not recognized, may impact the results, although propensity score-matching is one of the strongest methods to control confounding factors. Based on the E value, the observed higher all-cause mortality in patients with RA could be explained by an unmeasured confounder associated with both RA and mortality by a risk ratio of 2.4-fold each, above and beyond the measured confounders, but weaker confounding could not do so [14]. Smoking is a potential residual confounder in our study as it is associated with both RA and coronary artery disease. Even so, we consider it is unlikely that smoking alone would explain the main findings in our study.

## Conclusion

In conclusion, our study demonstrated that RA is independently associated with a poorer prognosis in coronary artery disease treated with CABG. Preoperative corticosteroid use and longer RA disease duration are additional risk factors for increased mortality. RA patients might benefit from optimized antirheumatic medication and secondary prevention of cardiovascular disease after CABG.

## Supplementary Material

Supplemental Material

## Data Availability

Authors are not allowed to provide data to third parties due to national legislation and regulations. Please see www.findata.fi/en/ for details.
