# Supplementary material for: Long-term outcomes after coronary artery bypass surgery in patients with rheumatoid arthritis
Source: Ann Med. 2021 Aug 31;53(1):1512–9. doi: 10.1080/07853890.2021.1969591 (PMC8409967; doi:10.1080/07853890.2021.1969591)
Supplement: Supplemental Material [file IANN_A_1969591_SM3549.zip › Supplemental files/Suppl_Table_1_CABG_RA_Malmberg.docx]

|  | | **ATC-codes** |
| --- | --- | --- |
| **ADP-inhibitor** | | B01AC04 , B01AC05, B01AC22, B01AC24 |
| **Anticoagulant** | | B01AA, B01AE, B01AF |
| **Antidiabetic** | | A10 |
|  | **Insulin** | A10A |
|  | **Non-insulin** | A10B |
| **ACEi or ARB** | | C09, C10BX04, C10BX06, C10BX07, C10BX10, C10BX11, C10BX12, C10BX13, C10BX14, C10BX15 |
| **Antiarrhythmic** | | C01B |
| **Beta-blocker** | | C07, C09BB, C09DA, C09BX02, C09DX05 |
| **Ca-blocker** | | C08, C07FB, C09BB, C09DB, C09DX01, C09DX03, C09DX06, C09XA53, C09XA54, C10BX03, C10BX07, C10BX09, C10BX11, C10BX14 |
| **Corticosteroid** | | H02AB |
| **Digitalis** | | C01A |
| **Diuretic** | | C03, C02L, C07B, C07C, C07D, C08G, C09BA, C09DA, C09BX01, C09BX03, C09DX01, C09DX03, C09DX05, C09DX06, C10BX13 |
| **Nitrate** | | C01DA |
| **Statin** | | C10AA, C10BA, C10BX |

**Supplement Table 1.** Anatomical Therapeutic Chemical Classification (ATC) codes for studied prescription drugs. ADP = adenosine diphosphate, ACEi = Angiotensin-converting-enzyme inhibitor, ARB = angiotensin receptor blocker.
