# Supplementary material for: Long-term outcomes after coronary artery bypass surgery in patients with rheumatoid arthritis
Source: Ann Med. 2021 Aug 31;53(1):1512–9. doi: 10.1080/07853890.2021.1969591 (PMC8409967; doi:10.1080/07853890.2021.1969591)
Supplement: Supplemental Material [file IANN_A_1969591_SM3549.zip › Supplemental files/Suppl_Table_2_CABG_RA_Malmberg.docx]

|  | **Statin intensity** | | |
| --- | --- | --- | --- |
|  | **Low** | **Moderate** | **High** |
|  | **mg** | **mg** | **mg** |
| **Atorvastatin** | - | 10 – 39 | ≥ 40 |
| **Fluvastatin** | < 80 | ≥ 80 | - |
| **Lovastatin** | < 40 | ≥ 40 | - |
| **Pravastatin** | < 40 | ≥ 40 | - |
| **Rosuvastatin** | - | 5 – 19 | ≥ 20 |
| **Simvastatin** | < 20 | 20 – 79 | ≥ 80 |

**Supplement Table 2.** Definitions for intensity of statin therapy. Intensity of therapy after coronary artery bypass grafting surgery was determined from first drug purchase after hospital discharge.
