# Supplementary material for: Long-term outcomes after coronary artery bypass surgery in patients with rheumatoid arthritis
Source: Ann Med. 2021 Aug 31;53(1):1512–9. doi: 10.1080/07853890.2021.1969591 (PMC8409967; doi:10.1080/07853890.2021.1969591)
Supplement: Supplemental Material [file IANN_A_1969591_SM3549.zip › Supplemental files/Suppl_Table_3_CABG_RA_7_21_rev_AnnMed.docx]

|  | | **Univariable** | | **Multivariable** | |
| --- | --- | --- | --- | --- | --- |
| **Variable** | | **HR (95% CI)** | **P-value** | **HR (95% CI)** | **P-value** |
| **Age (per 10-year increment)** | | 1.71 (1.39-2.10) | <0.0001 | 1.67 (1.33-2.11) | <0.0001 |
| **Female sex** | | 0.87 (0.63-1.21) | 0.410 | 0.78 (0.55-1.11) | 0.163 |
| **Atrial fibrillation** | | 1.91 (1.30-2.80) | 0.001 | 1.49 (0.98-2.26) | 0.061 |
| **Cerebrovascular disease** | | 1.36 (0.78-2.35) | 0.275 | 1.35 (0.75-2.42) | 0.322 |
| **Chronic pulmonary disease** | | 2.02 (1.41-2.89) | 0.0001 | 1.70 (1.15-2.51) | 0.007 |
| **Diabetes** | | 1.65 (1.16-2.33) | 0.005 | 1.88 (1.29-2.75) | 0.001 |
| **Heart failure** | | 1.87 (1.32-2.65) | 0.001 | 1.47 (1.00-2.14) | 0.048 |
| **Hypertension** | | 1.30 (0.95-1.77) | 0.108 | 0.93 (0.65-1.32) | 0.676 |
| **Malignancy** | | 1.46 (0.83-2.58) | 0.188 | 1.01 (0.54-1.89) | 0.986 |
| **Myocardial infarction*** | | 1.29 (0.93-1.79) | 0.124 | 1.32 (0.92-1.88) | 0.133 |
| **Peripheral vascular disease** | | 1.43 (0.90-2.29) | 0.134 | 0.97 (0.57-1.63) | 0.896 |
| **Psychotic disorder** | | 0.46 (0.07-3.29) | 0.440 | 0.96 (0.13-7.11) | 0.968 |
| **Renal disease** | | 4.28 (2.56-7.15) | <0.0001 | 3.42 (1.88-6.24) | <0.0001 |
| **Type of bypass graft** | |  | 0.011 |  | 0.173 |
|  | **Only arterial** | Reference |  | Reference |  |
|  | **Only venous** | 1.96 (1.07-3.58) | 0.030 | 1.49 (0.69-3.25) | 0.312 |
|  | **Arterial and venous** | 0.90 (0.59-1.35) | 0.601 | 0.83 (0.42-1.64) | 0.591 |
| **Number of grafted anastomoses** | |  | 0.902 |  | 0.989 |
|  | **1** | Reference |  | Reference |  |
|  | **2** | 0.92 (0.50-1.67) | 0.778 | 0.96 (0.43-2.10) | 0.908 |
|  | **3** | 0.80 (0.48-1.33) | 0.391 | 0.99 (0.45-2.16) | 0.975 |
|  | **4** | 0.80 (0.47-1.34) | 0.397 | 0.91 (0.39-2.09) | 0.818 |
|  | **≥5** | 0.82 (0.46-1.47) | 0.506 | 0.84 (0.34-2.10) | 0.712 |
| **Seropositivity** | | 1.16 (0.79-1.70) | 0.448 | 1.03 (0.69-1.57) | 0.874 |
| **Per oral corticosteroid usage** | | 1.48 (1.08-2.02) | 0.014 | 1.44 (1.03-2.01) | 0.032 |
| **Duration of RA**  **(per 5-year increment)** | | 1.02 (1.01-1.03) | 0.003 | 1.09 (1.02-1.17) | 0.009 |
| **Surgical center (n=8)** | |  | 0.530 |  | 0.160 |

* Prior or acute

**Supplement Table 3.** Association baseline features with long-term mortality after coronary artery bypass in patients with rheumatoid arthritis (n=378). Results of univariable and multivariable Cox models. HR = Hazard ratio. RA = rheumatoid arthiritis.
